# Navigating Brain Metastases: Unveiling the Potential of 3-Tesla Intraoperative Magnetic Resonance Imaging

**DOI:** 10.3390/cancers16162774

**Published:** 2024-08-06

**Authors:** Ghaith Altawalbeh, Maria Goldberg, Michel Gustavo Mondragón-Soto, Chiara Negwer, Arthur Wagner, Jens Gempt, Bernhard Meyer, Amir Kaywan Aftahy

**Affiliations:** 1 Department of Neurosurgery, School of Medicine, Klinikum Rechts der Isar, Technical University Munich, 81675 Munich, Germanybernhard.meyer@tum.de (B.M.); kaywan.aftahy@tum.de (A.K.A.); 2 Department of Neurosurgery, National Institute of Neurology and Neurosurgery, Mexico City 14269, Mexico; mmondragon@innn.edu.mx; 3 Department of Neurosurgery, University Medical Center Hamburg-Eppendorf, 20246 Hamburg, Germany

**Keywords:** intraoperative MRI (iMRI), brain metastases, brain cancer, quality of life

## Abstract

**Simple Summary:**

This study explores the use of intraoperative magnetic resonance imaging (iMRI) in the surgical resection of brain metastases (BMs). The research aims to determine how iMRI can improve the precision of tumor removal, thereby enhancing patient outcomes. By providing real-time, high-resolution images during surgery, iMRI assists surgeons in achieving more complete tumor resection, potentially reducing recurrence rates and improving survival chances. Integrating iMRI into surgical protocols helps preserve neurological functions, especially for patients with BM in critical brain areas. This study highlights the safety and effectiveness of iMRI in neurosurgery and suggests its broader adoption for the better management of BMs.

**Abstract:**

Intraoperative magnetic resonance imaging (iMRI) has witnessed significant growth in the field of neurosurgery, particularly in glioma surgery, enhancing image-guided neuronavigation and optimizing the extent of resection (EOR). Despite its extensive use in the treatment of gliomas, its utility in brain metastases (BMs) remains unexplored. This study examined the effect of iMRI on BM resection. This retrospective study was conducted at the neurosurgical center of the University Hospital of the Technical University of Munich and involved 25 patients with BM who underwent resection using 3-Tesla iMRI between 2018 and 2022. Volumetric measurements of the resected contrast-enhancing metastases were performed using preoperative, intraoperative, and postoperative MRI images. The Karnofsky Performance Score (KPS) and neurological status of the patients were assessed pre- and postoperatively. Local recurrence and in-brain progression were reported in patients who underwent follow-up MRI at 3 and 6 months postoperatively. In this cohort (*n* = 25, mean age 63.6 years), non-small-cell lung cancer (NSCLC) was the most common origin (28%). The mean surgical duration was 219.9 min, and that of iMRI was 61.7 min. Indications for iMRI were primarily associated with preoperative imaging, suggesting an unclear entity that is often suspicious for glioma. Gross total resection (GTR) was achieved in 21 patients (84%). Continued resection was pursued after iMRI in six cases (24%), resulting in an improved EOR of 100% in five cases and 97.6% in one case. Neurological status postoperatively remained stable in 60%, improved in 24%, and worsened in 16% of patients. No wound healing or postoperative complications were observed. Among the thirteen patients who underwent follow-up MRI 3 months postoperatively, one patient showed local recurrence at the site of resection, and seven patients showed in-brain progression. Of the eight patients who underwent a 6-month follow-up MRI, two showed local recurrence, while three exhibited in-brain progression. The observed favorable profiles of GTR, coupled with the notable absence of wound-healing problems and acute postoperative complications, affirm the safety and feasibility of incorporating iMRI into the neurosurgical workflow for resecting BM with specific indications. The real-time imaging capabilities of iMRI offer unparalleled precision, aiding meticulous tumor delineation and informed decision-making, ultimately contributing to improved patient outcomes. Although our experience suggests the potential benefits of iMRI as a safe tool for enhancing EOR, we acknowledge the need for larger prospective clinical trials. Comprehensive investigations on a broader scale are imperative to further elucidate the specific indications for iMRI in the context of BMs and to study its impact on survival. Rigorous prospective studies will refine our understanding of the clinical scenarios in which iMRI can maximize its impact, guiding neurosurgeons toward more informed and tailored decision-making.

## 1. Introduction

The treatment landscape for central nervous system (CNS) malignancies has significantly evolved and become increasingly complex and multimodal, necessitating a multidisciplinary approach [1,2,3,4,5]. At institutions where a multidisciplinary board is available, it can help optimize the coordination of care and maximize resources, as well as the involvement of subspecialists in management decisions. The main objective of neuro-oncological neurosurgery is to avoid local recurrence and simultaneously decrease neurological deficits secondary to the procedure [6]. Multiple tools have been developed to increase the reliability of navigation and boost GTR rates. Some conservative techniques include morphological imaging through intraoperative ultrasound, optical coherence tomography, and intraoperative magnetic resonance imaging (MRI), which are currently considered the gold standards for intraoperative techniques [7].

Advances in targeted therapy and immunotherapy have resulted in a high prevalence of metastatic cancer patients acquiring good systemic disease control, often leaving the CNS as the only site of uncontrolled disease. The presence of isolated BM in patients without systemic progression has changed the approach to BM management. Unlike glioma surgery, where the main goal has been centered on the ability to maximize EOR [8], in BM treatment, traditionally, the approach to CNS disease has relied more on the effect of systemic control, which leverages the role of surgery and focused radiation. Thus, it is important to consider reliable resources that may optimize postoperative oncological outcomes.

Aggressive surgical approaches to BM are critical in the management of metastatic diseases. Hence, the use of adjuvant resources has increased EOR. Patients with high performance scores (KPS > 70) and well-controlled primary disease or with otherwise effective systemic treatment options were considered for surgical management [2]. Stereotactic radiosurgery (SRS) is a very valuable option for patients with multiple BMs. Surgery is a treatment modality for symptomatic BMs [2,9]. However, achieving maximal cytoreduction has been associated with better outcomes, specifically improving overall survival [10,11]. This could also advance the early assessment of patients with BM to make prompt postoperative decisions [12]. Being able to perform a safer surgical resection could allow a wider selection of patients eligible for surgical treatment, including those with a poorer KPS [13], larger volume of metastases, and lesions that include highly eloquent areas.

BMs are the most common type of intracranial tumors in adults, occurring approximately 10 times more frequently than primary malignant brain tumors [14]. Improved surveillance, effective systemic therapy, and an aging population contribute to the increasing incidence of BM [15]. This poses a significant clinical challenge and highlights the need for effective management strategies.

The aggressive resection of surgically accessible BM correlates with improved functional status and higher survival rates. This has been reported, especially when correlated with a lower tumor resting volume [10,11]. This process may mitigate the rapid tissue infiltration by the tumor. Neuronavigation has proven to be an effective tool during oncological neurosurgery, substantially improving accuracy and EOR. However, it has some limitations, such as an inability to provide real-time intraoperative brain images and a susceptibility to brain shifts, which could negatively impact patient outcomes [16]. Brain shifts are related to the loss of cerebrospinal fluid (CSF), brain dependency, edema, and tumor removal, resulting in inaccuracies during resection [17].

iMRI may be used to determine the extent of residual tumor burden and provide updated navigational data [18]. It has emerged as a valuable tool in neurosurgery to overcome these challenges, enabling a real-time visualization of the tumor and adjacent eloquent structures, thus enhancing the precision of tumor removal [19]. The use of iMRI has increased over the past decade, especially in glioma surgery [1]. It can update image-guided neuronavigation intraoperatively and may improve EOR. However, several factors may limit the use of this imaging modality, including rates of perioperative infection, transoperative stroke, and surgical bleeding.

In a previous report [20], after additional resection after iMRI, 40.5% of glioma patients who underwent re-resection showed new surgery-related deficits, which persisted in 24.3% of the cases. Among the cases without additional resection after iMRI, new motor deficits were reported in 23.5% of cases, with 9.2% of cases persisting in the long term. However, no significant long-term differences were found in language and motor deficits between patients with and without additional resection.

The available evidence has been explored widely in recent years for primary brain tumors; however, the literature discussing the effectiveness of iMRI for the resection of BM remains limited to small studies and case reports. To resolve this inconsistency, we performed one of the first available studies on tumor resection using iMRI for BM. The primary objective of our study was to study the feasibility of iMRI and to determine its impact on clinical outcomes and EOR. The impact on survival requires further investigations. By assessing our data, we aimed to provide valuable insights that could contribute to clinical decision-making regarding the management of these complex clinical entities. This technique could facilitate larger and safer tumor resections in patients with BM.

## 2. Materials and Methods

### 2.1. Study Population and Data Collection

In February 2018, a 3-Tesla intraoperative magnetic resonance imaging (iMRI) system was integrated into the operating room at the University Hospital of the Technical University of Munich (Klinikum Rechts der Isar). A retrospective analysis was conducted to identify twenty-five patients who underwent a surgical resection of BM between 2018 and 2022 from our clinic records and met the inclusion criteria (a histopathological diagnosis of BM; pre-, intra-, and postoperative MRI; and tumor resection apart from brain tumor biopsy). Patients who underwent biopsies, those with unclear histological findings, or those who underwent surgery for the resection of recurrent BM were excluded.

Patient medical records including age at operation, sex, tumor localization, the number of BMs, the date of surgery, and preoperative and postoperative KPS. The duration of the operation and iMRI, primary tumor type, indication for iMRI, preoperative tumor volume, intraoperative residual tumor volume, residual tumor volume postoperatively, EOR, postoperative neurological status, postoperative compilations, and recurrence in the resection site or in-brain progression in the follow-up MRI assessments at 3 months and 6 months postoperatively were evaluated.

### 2.2. Surgical Procedure and Imaging Analysis

The surgical approach aims to achieve extensive tumor removal while protecting the eloquent area of the brain. It was performed using preoperative and intraoperative navigation techniques with iMRI (3T MR scanner Ingenia, Philips Medical System, Netherlands B.V., Eindhoven, The Netherlands). The indications for surgical treatment were based on the mass effect, bleeding, the development of new neurological deficits, and uncertainty regarding the nature of the tumor. Contrast-enhancing tumor volumes were manually segmented and analyzed by experienced faculty members using Origin software (SmartBrush, version 3.1, Brainlab AG, Munich, Germany).

Before incision, craniotomies and surgical approaches were planned using a stereotactic navigation system (Brainlab AG, Munich, Germany). Preoperative mapping data of motor and speech areas were loaded into the neuronavigation system. Motor-evoked potential monitoring was performed in patients with BM in eloquent motor areas. Microsurgical resection was performed using bipolar cautery, suction, and an ultrasonic tumor aspiration device (Integra Life Sciences, Princeton, NJ, USA). IMRI was performed when the surgeon deemed that the initially intended volume of the tumor had been resected. All neurosurgeons were trained in using iMRI and the procedures were performed according to our hospital’s standard protocol. This included standard draping and safety procedures that were completed before performing iMRI studies. All procedures were performed in iMRI suites with an adjacent 3-Tesla MRI machine in the operating room. A 3D 1 mm T1-weighted series with gadolinium was performed in each case. The iMRI scans were loaded into the neuronavigation system, and the iMRI images were registered. Further tumor resection was performed as indicated. In cases of complete tumor resection or reaching intended resection, the operation was terminated after an extensive inspection of the resection cavity, cautery, the closure of the dura, the replacement of the scalp, and wound closure.

### 2.3. Postoperative Protocol

Neurological status was extensively assessed immediately after the surgical procedure. All patients underwent postoperative 3-Tesla MRI within the first 48 h of surgery. A regular inspection of the wound was performed throughout the hospital stay and before the patient was discharged. An interdisciplinary tumor board within our institution, comprising neurosurgeons, neuro-radiologists, neuro-oncologists, oncologists, neuropathologists, and radiation therapists, was established to determine the optimal treatment strategy for patients with BM. Adjuvant treatments were tailored to histopathological findings and adhered to established standards of care and interdisciplinary treatment protocols, including chemotherapy, radiotherapy, and combined treatment regimens.

### 2.4. Institutional Review Board Statement

The study and data collection were approved by the Ethics Committee of the Technical University of Munich (No. 5626:12, 10.10.2020) and adhered to the ethical standards outlined in the Declaration of Helsinki.

## 3. Results

### 3.1. Patient Demographics and Baseline Characteristics

This cohort comprised 12 male patients (48%) and 13 female patients (52%) with a mean age of 63.6 years (SD = 11.9 years). The mean duration of the surgical procedure was 219.9 min (SD = 74.5 min) and the mean iMRI duration was 61.7 min (SD = 18 min). Non-small-cell lung cancer (NSCLC) was histologically the most common origin (N = 7, 28%). Indications for iMRI were primarily associated with preoperative imaging, suggesting an unclear entity which is often suspicious for glioma. Other indications include a large volume of BM and its localization in eloquent brain areas. The most common location was parietal (N = 9, 36%). Frontal metastasis was the second most frequent (N = 7; 28%). Other BMs were located temporal (N = 4; 16%), occipital (N = 3; 12), thalamic (N = 1; 4%), and sellar (N = 1; 4%). Fourteen of the resected BMs were in the left hemisphere and eleven were in the right hemisphere. Patient demographics and baseline characteristics are summarized in Table 1.

### 3.2. Impact of iMRI on Increasing Excess of Resection (EOR)

The mean preoperative tumor volume was 18.4 cm^3^, with tumors ranging from 2.7 cm^3^ to 62.8 cm^3^. At the time of intraoperative imaging, the mean residual tumor volume was 1.14 cm^3^, with intraoperative residual volumes ranging from 0 to 8 cm^3^. The mean residual tumor volume postoperatively decreased to 0.67 cm^3^ after continued resection after iMRI.

**Figure 1:**
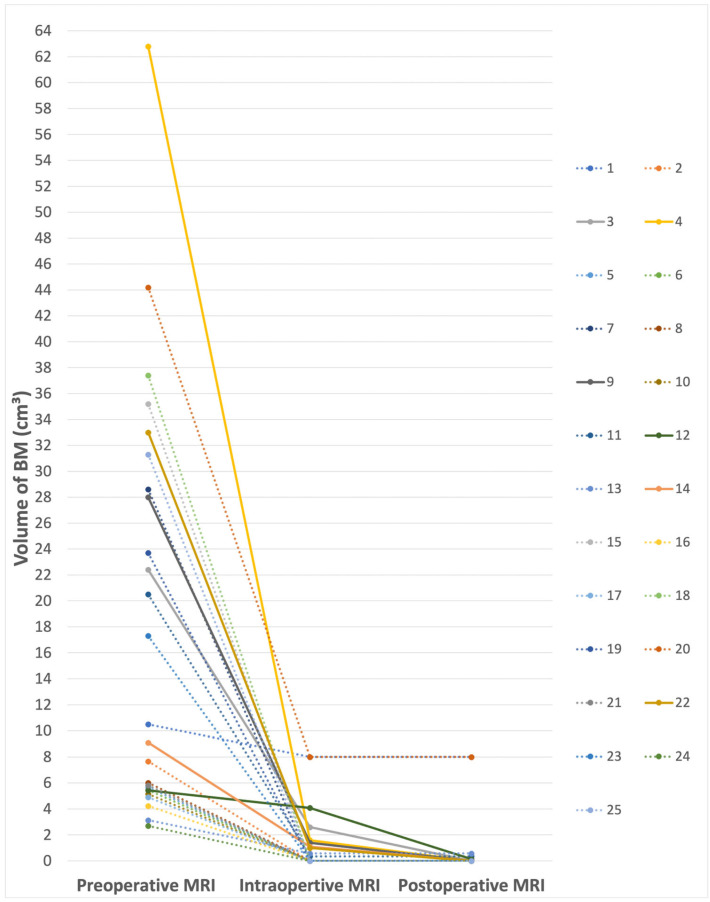
Volumes of the resected BM for each patient in intra- and postoperative MRI after subsequent resection. Patients who underwent further resection after iMRI are marked with solid lines.

In 15 patients (60%), gross total resection was seen in the iMRI. Subtotal resection (STR) was performed in 10 cases (40%). Intraoperative MRI provided real-time feedback, aiding in the identification of residual tumor burden (RTB) and eloquent areas, and facilitating adjustments in the surgical strategy. This resulted in six cases (24%) in which subsequent resection was pursued based on iMRI feedback. This led to improving EOR to 100% in five cases and to 97.6% in one case. The mean EOR increased from 91.06% (SD = 20.9%) to 95.4% (SD = 15.7%) after further resection after iMRI. The volumes of the resected BMs for each patient on preoperative, intraoperative, and postoperative MRI are shown in Figure 1 and Table 2. EOR with and without iMRI is demonstrated in Table 2.

### 3.3. Postoperative Outcome and Follow-Up

The mean preoperative KPS was 82% (SD = 14.1%), with a slight decrease to 78.8% (SD = 21.4%) postoperatively. This decrease can be attributed primarily to two factors. Firstly, one patient, as detailed in Section 3.4., died in the postoperative course due to pneumonia related to an underlying lung tumor. This severe complication significantly impacted the overall KPS average. Secondly, while 60% of patients (*n* = 15) maintained their preoperative neurological status, and 24% (*n* = 6) experienced partial or complete relief from preoperative deficits, 16% (*n* = 4) displayed a worsening of neurological deficits immediately postoperatively (Table 2). Of the six patients who underwent further resection after iMRI, only one individual exhibited a postoperative worsening of neurological deficits, resulting in a decrease in KPS from 70% at admission to 50% at discharge. The patient exhibited a marked improvement of the neurological status after rehabilitation. None of the patients experienced wound healing issues.

Thirteen patients underwent follow-up MRI 3 months postoperatively, revealing one case of local recurrence at the resection site and in-brain progression in seven patients unrelated to a resected metastasis. Among the eight patients who underwent a 6-month follow-up MRI, two demonstrated local recurrence, while three exhibited in-brain progression. The retrospective recruitment of additional data was hindered by some patients receiving follow-up at other clinics, leading to challenges in data collection due to disrupted communication links with these patients.

### 3.4. Complications and Adverse Events

In patient No. 1 with a hepatocellular metastasis in the skull base, resection had to be interrupted due to strong bleeding. This led to interrupting the operation and having an outlining EOR of 23.81% (preoperative tumor volume: 10.5 cm^3^; residual tumor volume postoperatively: 8 cm^3^). The patient underwent a second surgery to further debulk the tumor.

Regarding patient No. 20, only a partial resection of the metastasis was intended. The patient had a large symptomatic BM from bronchial carcinoma located in the right temporal lobe with an invasive nature and proximity to the middle cerebral artery and brainstem circumferential vessels. Therefore, a complete resection of the metastasis was not performed.

Patient No. 7 exhibited severe left-sided hemiparesis postoperatively. During further postoperative course, there was a progressive clinical deterioration with dyspnea due to pneumonia, as well as a progression of the primary lung tumor, causing the compression of the right main bronchus and atelectasis of the right lower lobe. Additionally, there was a progression of liver metastases and adrenal gland metastases. The treatment goal was changed to palliative care and the patient passed away a few weeks postoperatively.

## 4. Discussion

The series presented in this manuscript is among the first to document the usefulness of iMRI for BM resection. A total of 25 patients were treated with this modality, with an acceptable increase in operative time while using the iMRI, as well as a tolerable number of cases that required a complementary resection in addition to the initial one (24%), improving to a complete resection in a total of five patients, and only one case with 97.6% of complete resection. The limited long-term follow-up, complicated by patients receiving care at other facilities, impacted our ability to thoroughly assess the long-term outcomes secondary to the invasive and systemic nature of the underlying disease in these patients. The data presented may only be compared to the data from patients with glioma, although the natural history of both diseases tends to differ.

However, high-quality evidence regarding the use of iMRI in glioma surgery remains limited. The effects of image-guided resection on overall survival (OS), progression-free survival (PFS), and quality of life in patients with glioma have been reported; however, a functional comparison has not been clearly demarcated [21,22]. The first clinical trial of iMRI in patients with gliomas was reported by Senft et al. [23] where more patients in the intraoperative MRI group underwent complete tumor resection (96%) than in the control group (68%, *p* = 0.023). The postoperative rates of new neurological deficits did not differ between the groups; only one patient died within 6 months after surgery. Patients who underwent complete tumor resection had longer PFS than those with residual tumors (median, 226 vs. 98 days, *p* = 0.003).

In a clinical trial [24], GTR was 83.85% in the iMRI group and 50% in the control group (*p* < 0.001). The median PFS was 65.23 months in the iMRI group and 61.01 in the control group (*p* = 0.0202). In their series, a residual tumor volume < 1.0 cm^3^ decreased the risk of survival (mPFS: 18.99 vs. 0.43 months, *p* = 0.0055; mOS: 29.77 vs. 18.1 months, *p* = 0.0042). In another clinical trial, GTR was finally achieved in glioma patients in 86.36% in the iMRI group versus 53.49% in the control group (*p* < 0.001). For PFS, there were four events (18.8%) in the iMRI group and six events (including five deaths; 40%) in the control group. OS was not included in the interim analysis. There were no differences in neurological deficits between the groups [25].

A meta-analysis performed by Golub et al. reported that surgical resection with the guidance of iMRI was superior to resection with neuronavigation alone (OR 4.99, 95% CI 2.65–9.39, *p* < 0.001), although their study did not explore the influence of the use of intraoperative ultrasound and fluorescence-guided resection.

Despite the prolonged surgery time and transfer in non-sterile MRI-scanner, no surgical site infections (SSIs) were reported in the postoperative course of this study. A previous retrospective trial conducted at our center did not reveal any increased risk of SSI after utilizing iMRI for the resection of brain tumors [26].

Various iMRI modalities have been developed in recent years. Initial experiences involving a trans-sulcal tubular retractor approach in 10 patients, coupled with a tubular retractor system, reported a subtotal resection in 6 out of 10 cases (60%), with additional resection in 5 of 6 cases (83%), reducing the subtotal resection rate to 2 out of 10 cases (20%) [20]. Owing to their close relationship with eloquent structures, 30% of patients experienced new postoperative neurological deficits, of which two were transient. Among them, four patients had brain metastases.

Other possible intraoperative techniques and technologies, as mentioned before, include the use of navigated ultrasound. This technique has been reported to have high concordance with navigation and may be readily available for use. Limitations include a lower image yield, lower image resolution when compared to MRI, and accuracy depending on user experience [27]. Studies need to be performed to clarify its validity compared with iMRI.

The decision to incorporate intraoperative MRI into our patient cohort was based on specific clinical considerations that enhanced the strategic surgical approach in these cases. IMRI proved instrumental in cases with large tumor sizes, localization in eloquent areas of the brain, or areas that were surgically challenging to access, as well as in instances characterized by unclear imaging findings or suspected differential entities. The real-time, high-resolution imaging provided by iMRI is crucial for precise tumor delineation and facilitates optimal surgical decision-making. This technology supports the neurosurgical team in making informed decisions during surgery, particularly when dealing with tumors that are adjacent to crucial neurovascular structures or those whose boundaries are not clearly defined on preoperative imaging [6,15,16]. IMRI promises to become as critical in the surgical resection of BMs as it is in glioma surgery [1,28]. Its ability to provide real-time, high-definition imaging enables surgeons to distinguish between tumor tissue and normal brain tissue with exceptional clarity, ensuring a more precise removal of metastatic lesions while maximizing the preservation of healthy brain tissue, thereby optimizing patient outcomes in a way previously reserved for glioma treatment protocols.

The retrospective nature of this analysis and the absence of a comparative group restricted our ability to robustly determine potential risk factors associated with the use of iMRI in the treatment of BM. Additionally, the small sample size may have limited the comprehensiveness of complication reporting. Another significant constraint was the inability to present survival data; the retrospective design and lost communication links with several patients, who received follow-up care at other facilities, hindered comprehensive long-term outcome assessment. This lack of detailed follow-up data notably affects the study’s capacity to comprehensively assess the long-term impact of iMRI on survival and disease progression.

Despite these limitations, this study stands as one of the first to document the utility of iMRI in the resection of BM, potentially serving as a foundational reference for future research. By outlining the conditions under which iMRI was employed and noting the specific scenarios that benefited from its use, this research may help neurosurgeons better identify proper indications for intraoperative MRI in future practice.

## 5. Conclusions

In conclusion, the integration of intraoperative MRI into the resection of BMs presents a promising enhancement to surgical outcomes. Leveraging real-time imaging capabilities has demonstrated improvements in EOR. Importantly, this study has shown that iMRI serves as a safe tool for patients, contributing to more precise surgical interventions without increasing the risk of significant complications. However, it is crucial to recognize the potential challenges and nuances associated with this technology. Further studies are warranted to refine its application and establish a more comprehensive understanding of its impact in the management of brain metastases, particularly concerning long-term survival and quality of life.

## Figures and Tables

**Table 1:** Patient demographics and baseline characteristics.

No.	Age at Operation	Sex	Duration of Operation (min)	Duration of iMRI (min)	Indication of iMRI *	Histology	Localization
1	37	Male	450	60	2	Hepatocellular carcinoma (HCC)	Sellar
2	65	Male	170	40	1	Urothelial carcinoma	Temporal
3	58	Female	320	40	2	Non-small-cell lung cancer (NSCLC)	Occipital
4	65	Male	290	60	1 + 3	Cancer of unknown primary (CUP)	Frontal
5	73	Male	180	80	2	Ovarian cancer	Parietal
6	47	Male	136	40	2	Seminoma	Precentral
7	63	Male	280	45	1	Non-small-cell lung cancer (NSCLC)	Central
8	69	Male	116	70	2	Colorectal cancer (CRC)	Parietal
9	67	Female	220	75	3	Non-small-cell lung cancer (NSCLC)	Parietal
10	55	Female	110	40	1	Non-small-cell lung cancer (NSCLC)	Parietal
11	79	Male	180	80	1	Melanoma	Parietal
12	52	Female	240	65	2	Breast cancer	Thalamic
13	67	Female	180	60	2	Colorectal cancer (CRC)	Postcentral
14	55	Male	300	75	1	Urothelial carcinoma	Frontal
15	80	Male	210	60	3	Melanoma	Frontal
16	66	Female	180	80	1	Esophageal cancer	Postcentral
17	42	Female	240	40	1	Breast cancer	Temporal
18	73	Female	290	60	1	Breast cancer	Occipital
19	47	Female	230	105	2	Cervical cancer	Postcentral
20	60	Female	210	70	3	Small-cell lung cancer (SCLC)	Temporal
21	71	Female	180	60	2	Non-small-cell lung cancer (NSCLC)	Postcentral
22	76	Male	260	90	1	Prostate cancer	Temporal
23	70	Female	190	40	1	Non-small-cell lung cancer (NSCLC)	Occipital
24	80	Female	147	40	1	Non-small-cell lung cancer (NSCLC)	Frontal
25	74	Male	188	67	3	Renal-cell cancer (RCC)	Frontal

* 1: Suspicious imaging (e.g., for glioma); 2: Unfavorable or eloquent location; 3: Size of BM.

**Table 2:** Volume of the resected BM in preoperative, intraoperative, and postoperative MRI in addition to EOR with and without iMRI.

No.	Preoperative Tumor Volume (cm^3^)	Intraoperative Residual Volume (cm^3^)	Postoperative Residual Volume (cm^3^)	Further Resection after iMRI	EOR without iMRI (%)	EOR with iMRI (%)	KPS at Admission	KPS at Discharge
1	10.5	8	8	No	23.81	23.81	70	90
2	7.66	0	0	No	100	100	90	90
3	22.4	2.6	0	Yes	88.39	100	60	60
4	62.8	1.59	0	Yes	97.47	100	90	90
5	5.66	0	0	No	100	100	90	90
6	5.44	0	0	No	100	100	90	80
7	28.6	0	0	No	100	100	40	0
8	6.01	0	0	No	100	100	100	100
9	28	1.4	0	Yes	95	100	90	90
10	5.15	0	0	No	100	100	100	100
11	20.5	0.35	0.35	No	98.29	98.29	90	90
12	5.43	4.08	0.13	Yes	24.86	97.61	70	50
13	3.13	0.57	0.57	No	81.79	81.79	80	90
14	9.1	1.1	0	Yes	87.91	100	90	90
15	35.2	0	0	No	100	100	70	80
16	4.23	0	0	No	100	100	90	50
17	4.9	0	0	No	100	100	90	90
18	37.4	0	0	No	100	100	80	80
19	23.7	0	0	No	100	100	70	80
20	44.2	8	8	No	81.9	81.9	90	90
21	5.82	0	0	No	100	100	90	90
22	33	1	0	Yes	96.97	100	80	80
23	17.3	0	0	No	100	100	90	90
24	2.7	0	0	No	100	100	90	60
25	31.3	0	0	No	100	100	60	80

## Data Availability

The original contributions presented in the study are included in the article, and further inquiries can be directed to the corresponding author.
